# Sex differences in the effect of fish-oil supplementation on the adaptive response to resistance exercise training in older people: a randomized controlled trial^1^,^2^

**DOI:** 10.3945/ajcn.116.140780

**Published:** 2016-11-16

**Authors:** Mariasole Da Boit, Rachael Sibson, Selvaraj Sivasubramaniam, Judith R Meakin, Carolyn A Greig, Richard M Aspden, Frank Thies, Stewart Jeromson, D Lee Hamilton, John R Speakman, Catherine Hambly, Arduino A Mangoni, Thomas Preston, Stuart R Gray

**Affiliations:** 3 Institute of Medical Sciences and; 4 School of Biological Sciences, University of Aberdeen, Aberdeen, United Kingdom;; 5 Exeter MR Research Centre, University of Exeter, Exeter, United Kingdom;; 6 School of Sport, Exercise and Rehabilitation Sciences and; 7 Medical Research Council Arthritis Research United Kingdom Centre for Musculoskeletal Ageing Research, University of Birmingham, Birmingham, United Kingdom;; 8 Faculty of Health Sciences and Sport, University of Stirling, Stirling, United Kingdom;; 9 Institute of Genetics and Developmental Biology, Chinese Academy of Sciences, Beijing, China;; 10 Department of Clinical Pharmacology, School of Medicine, Flinders University, Adelaide, Australia; and; 11 Scottish Universities Environmental Research Centre, University of Glasgow, Glasgow, United Kingdom

**Keywords:** aging, exercise, fatty acids, muscle, sarcopenia

## Abstract

**Background:** Resistance exercise increases muscle mass and function in older adults, but responses are attenuated compared with younger people. Data suggest that long-chain n–3 polyunsaturated fatty acids (PUFAs) may enhance adaptations to resistance exercise in older women. To our knowledge, this possibility has not been investigated in men.

**Objective:** We sought to determine the effects of long-chain n–3 PUFA supplementation on resistance exercise training–induced increases in muscle mass and function and whether these effects differ between older men and women.

**Design:** Fifty men and women [men: *n* = 27, mean ± SD age: 70.6 ± 4.5 y, mean ± SD body mass index (BMI; in kg/m^2^): 25.6 ± 4.2; women: *n* = 23, mean ± SD age: 70.7 ± 3.3 y, mean ± SD BMI: 25.3 ± 4.7] were randomly assigned to either long-chain n–3 PUFA (*n* = 23; 3 g fish oil/d) or placebo (*n* = 27; 3 g safflower oil/d) and participated in lower-limb resistance exercise training twice weekly for 18 wk. Muscle size, strength, and quality (strength per unit muscle area), functional abilities, and circulating metabolic and inflammatory markers were measured before and after the intervention.

**Results:** Maximal isometric torque increased after exercise training to a greater (*P <* 0.05) extent in the long-chain n–3 PUFA group than in the placebo group in women, with no differences (*P >* 0.05) between groups in men. In both sexes, the effect of exercise training on maximal isokinetic torque at 30, 90, and 240° s^−1^, 4-m walk time, chair-rise time, muscle anatomic cross-sectional area, and muscle fat did not differ (*P >* 0.05) between groups. There was a greater (*P <* 0.05) increase in muscle quality in women after exercise training in the long-chain n–3 PUFA group than in the placebo group, with no such differences in men (*P >* 0.05). Long-chain n–3 PUFAs resulted in a greater decrease (*P <* 0.05) than the placebo in plasma triglyceride concentrations in both sexes, with no differences (*P >* 0.05) in glucose, insulin, or inflammatory markers.

**Conclusion:** Long-chain n–3 PUFA supplementation augments increases in muscle function and quality in older women but not in older men after resistance exercise training. This trial was registered at clinicaltrials.gov as NCT02843009.

## INTRODUCTION

Older adults experience a loss of skeletal muscle mass termed sarcopenia (1). The mechanisms underlying sarcopenia remain to be fully established (2). Aging results not only in a loss of muscle mass but also a decrease in muscle strength (dynapenia). Several studies have shown that in older adults the decline in muscle strength is ∼2–4-fold more rapid than the reduction in muscle mass, indicating a decline in muscle quality (strength generated per unit of muscle mass) (3, 4). Resistance exercise is beneficial for maintaining and improving muscle mass, strength, and quality in older adults (5, 6). The magnitude of increase with exercise is, however, less than that seen in younger people and may differ between sexes (7, 8). Nutrition is frequently suggested to be a critical factor in determining the response to resistance exercise in older adults (9), and recent evidence has emerged to suggest that the fish oil–derived long-chain n–3 PUFAs, mainly EPA and DHA, may be important (10).

Indeed, early animal and cell work highlighted the potential of long-chain n–3 PUFAs (11–14). In humans, long-chain n–3 PUFA supplementation increases muscle mass and function in sedentary people (15–17). In addition, one study (18) investigated whether long-chain n–3 PUFAs enhance the adaptive response to the effects of resistance exercise in older adults. Increases in peak torque and the rate of torque development after 90 d resistance exercise were greater in older women supplemented with 2 g long-chain n–3 PUFAs/d. This study, however, included only women, was not placebo-controlled, and did not include muscle mass measurements. These drawbacks limit the wider applicability of these findings. A wealth of evidence has highlighted the potency of the placebo effect (19). Thus, whether this is a true or placebo effect and whether such an effect is seen in men both remain to be established. Indeed, in original epidemiologic work, demonstrating an association between fatty (rich in EPA/DHA) fish consumption and grip strength, each additional portion of fatty fish consumed per week was associated with a 1.8% gain in grip strength in women and only a 1.0% gain in men (20).

Therefore, we sought to determine the effects of long-chain n–3 PUFA supplementation on the adaptations in skeletal muscle mass, function, and systemic inflammation and whether these effects differed between men and women. Our hypothesis was that long-chain n–3 PUFA supplementation would enhance the adaptive responses in muscle mass and function to resistance exercise training in both men and women but that the effect would be greater in women than in men.

## METHODS

### Participants

**FIGURE 1:**
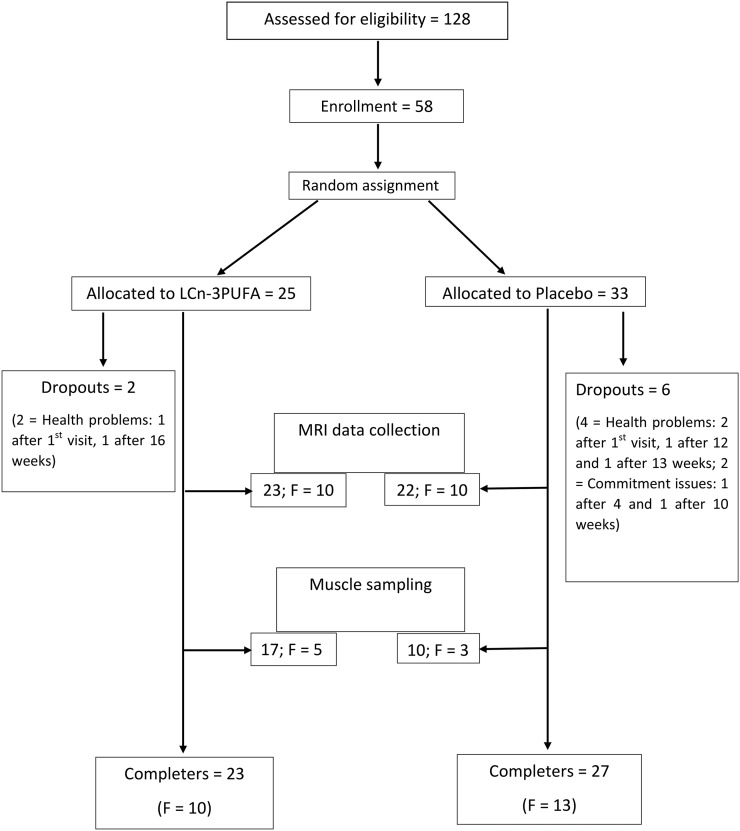
Flow diagram illustrating the participant progress through the phases of the study. F, female; LC, long chain.

Fifty-eight men and women were enrolled in the study (NCT02843009) between 2012 and 2014 (**Figure 1**). After being randomly assigned, 8 volunteers withdrew from the study; 50 participants (27 men and 23 women) completed it. Participants were recruited via posters and advertisements published in local newspapers and magazines. Inclusion criteria were being medically stable (i.e., free from cardiac illness, cancer arthritis, respiratory disease, metabolic disease, recent fractures, and loss of mobility) (21), not taking daily analgesia or nutritional supplements, and not participating in any resistance exercise training. Two participants were taking medications: 1 woman was prescribed angiotensin-converting enzyme inhibitors for mild hypertension and 1 man was prescribed allopurinol for gout. They were asked to continue taking their medication throughout the study. The study was approved by the University of Aberdeen College of Life Sciences and Medicine Ethics Review Board. Written consent was obtained after explaining the aims, risks, and potential discomfort associated with the study, which conformed to the Declaration of Helsinki. Data from the control group of this study was recently published in a separate manuscript investigating sex differences in response to resistance exercise alone (8).

### Study protocol

Upon entering the study, participants were randomly assigned (1:1 via a random number table) in a double-blind fashion to consume either 3 capsules long-chain n–3 PUFAs/d (3 × 1 g capsules giving 2.1 g EPA/d + 0.6 g DHA/d) (Barleans) or 3 identical-looking capsules of placebo (safflower oil: 3.0 g/d) (Barleans) every day for 18 wk. This dose was chosen on the basis of pilot data that demonstrated a beneficial effect on adaptations to strength in response to resistance training in older women (data not shown). At the end of the trial, the adherence of volunteers was evaluated by leftover pill count. Participants were asked to maintain their habitual diet and physical activity and to record any fish consumed in a diary for the duration of the study. Participants were also asked to refrain from exercise for 48 h before each study visit, and testing was carried out at the same time of the day. Physical activity levels were measured with use of an international physical activity questionnaire when the study commenced. At baseline and 18 wk, body mass, resting heart rate (HR)^14^, and blood pressure (BP) were measured. HR and BP were measured after 10 min seated rest with an automated sphygmomanometer. The mean of 3 measurements was used.

Resistance exercise training was performed by both groups twice a week for 18 wk. Each training session included 4 sets of 9 repetitions for each lower-body exercise: leg press, leg extension, leg curl, and calf press. The load for each exercise was set at 70% of the participants’ 1 repetition maximum. This was assessed for each exercise at baseline and every 6 wk, and the load was readjusted accordingly. All training sessions were supervised by a qualified instructor and scheduled at a time of day suitable for the participant.

### Measurements

The following measurements were made at baseline and at 18 wk.

#### Knee-extensor isometric and isokinetic torque

Maximal isometric torque of the knee-extensor muscles of the right leg was determined during a maximal voluntary contraction with the participant seated on a Biodex dynamometer with a knee angle of 73°. Participants were secured with the use of seatbelts, and the settings were recorded and reproduced during successive visits. Each maximal voluntary contraction was repeated ≥3 times, and the highest value was used for subsequent analysis. With the same seating position, maximal isokinetic torque of the knee extensors was measured at 30, 90, and 240°s^−1^, and the highest of 5 attempts was recorded. The range of motion for the isokinetic tests was 120 to 15°.

#### Short-performance physical battery test

The short-performance physical battery (SPPB) test consists of balance, walking speed, and timed chair-stand tests (22). The balance tests required participants to maintain a side-by-side, semi-tandem, and tandem stance for 10 s. All participants were able to complete the 3 balance tests for the full 10 s, so these data are not presented. The chair-stand test involved participants rising from a chair with their arms across their chest 5 times. This was repeated 3 times, and the fastest time was recorded for analysis. Participants were then instructed to complete 3 separate 4-m walks at the fastest pace possible, with the fastest time recorded for analysis.

#### MRI

Of the 50 participants who completed the intervention, 45 participants [*n* = 22 (10 women) in the placebo and *n* = 23 (10 women) in the long-chain n–3 PUFA group] were able to participate in MRI data collection. The other 5 participants were unable to undergo scans because 3 suffered from claustrophobia and 2 had metal implants. All scans were carried out on a Philips Achieva 3.0T whole-body MRI scanner with the use of a 16-channel sensitivity-encoding (SENSE XL Torso) coil, and muscle anatomic cross-sectional area (ACSA) was calculated as described previously (8). Muscle quality was calculated as torque (knee-extensor isometric strength) per unit ACSA.

#### Blood sampling

Fasted blood samples were collected from a vein in the antecubital fossa into vacutainers containing K^+^ EDTA, placed on ice, and processed within 30 min. Samples were centrifuged for 10 min at 4°C at 800 × *g*, and the erythrocytes and plasma were separated and stored at −80°C until analysis. Plasma glucose and triglycerides (TGs) were measured in duplicate with the use of a commercially available spectrophotometric assay following the manufacturer’s instructions (Randox). Insulin (Mercodia) and IL-6 and TNF-α (R&D Systems) were measured in duplicate with the use of commercially available ELISA assays according to the manufacturer’s instructions.

#### Fatty acid composition

Total lipids were extracted from erythrocytes and muscle tissue with chloroform:methanol (2:1 vol:vol) in the presence of 0.01% butylated hydroxytoluene to prevent fatty acid oxidation. Membrane phospholipids from the muscle tissue were separated by thin-layer chromatography with the use of a mixture of hexane:diethyl ether:acetic acid (90:30:1 vol:vol:vol). Fatty acid methyl esters were then prepared by incubation with 14% boron trifluoride in methanol at 80°C for 60 min and analyzed by gas chromatography as previously described (23).

### Muscle protein synthesis and p70s6k study

Twenty-seven participants (7 women) consented to take part in this voluntary substudy (i.e., several participants did not consent to undergo the muscle biopsy procedure). Because of the small numbers of participants in this substudy, no analysis of potential sex differences in response was done. To determine whether free-living muscle protein synthesis (MPS) differed between the placebo and long-chain n–3 PUFA groups, MPS was measured over a 4-d period (including 2 exercise sessions). To determine whether the acute resistance exercise induction of p70s6k activity differed between placebo and long-chain n–3 PUFA groups, p70s6k activity was measured before and 2 h after a training session in the last week of the intervention. The last week of training was chosen because the incorporation of long-chain n–3 PUFAs would be maximized at this point and to remove any of the contribution from the acute muscle-damage responses when beginning exercise training (24).

After collecting basal blood samples, participants were asked to consume a 200-mL (four 50-mL drinks) oral bolus of 70 atomic% deuterated water (D_2_O) (Goss Scientific) to label the body water pool. Resistance exercise sessions took place on days 2 and 4 after the bolus. Two 20-mL top-up doses of D_2_O were administered on days 3 and 4. Midstream urine samples were collected by the participant into a plastic container before the initial D_2_O bolus and daily thereafter and stored at −20°C for subsequent determination of body water enrichment. On day 4 after the administration of the D_2_O bolus and after an overnight fast, a resting muscle sample (∼70 mg) was collected after local anesthesia with the use of the microbiopsy technique (Bard Magnum; Bard Biopsy) from the vastus lateralis muscle of the right leg. Participants then performed a resistance exercise session as normal, and an additional muscle sample was collected 2 h after the cessation of exercise. Muscle tissue was rapidly cleaned from fat and connective tissue, washed in saline, frozen in liquid nitrogen, and stored at −80°C until further analysis.

The resting sample was used for the analysis of muscle fatty acid composition as described previously. The resting and 2-h postexercise samples were used for the analysis of p70s6k activity as previously described but with minor modifications (25), and the initial fasting blood sample, urine samples, and 2-h postexercise muscle sample were used for the MPS analysis (**Supplemental Methods**). These procedures for using D_2_O to measure MPS were developed in our previous work, and the single biopsy approach for quantifying MPS has previously been validated (26, 27). This measure of free-living MPS is an integration of basal MPS and the MPS response to the anabolic stimuli such as feeding and exercise.

### Statistical analysis

The primary outcome on which the required sample size was calculated to detect a difference at 18 wk in this study was knee-extensor maximal isometric torque. Based on a physiologically relevant difference of 34 N·m, which results in improved functional mobility (28), and a variability of ∼11% between individuals, 12 men and 12 women in each group would provide 80% statistical power at α = 0.05. Statistical analysis was performed with the use of SPSS version 22 (IBM) and Stata/MP version 14 (StataCorp LP). Data are presented as means ± SDs. All data were tested for normality and skewness before selecting the appropriate test. Univariable analyses with Student’s *t* test for normally distributed variables and the Mann-Whitney *U* test for skewed variables were used to assess differences, within each sex, in baseline characteristics between the long-chain n–3 PUFA and placebo groups. A 2-factor ANOVA was performed with baseline as a covariate to determine whether the change in variables over the 18 wk differed by sex or by group and whether a sex-by-group interaction was observed. When no interaction effect was observed, this was removed from the model. MPS was compared between long-chain n–3 PUFA and placebo groups via an independent *t* test, and sex differences were not tested because of the low number of participants. *P* < 0.05 was considered statistically significant.

## RESULTS

### Baseline participant characteristics

Baseline physical characteristics are presented in **Table 1**. Within each sex all baseline characteristics were similar (*P* > 0.05) between placebo and treatment groups. Participants’ baseline physical activity and weekly oily and nonoily fish consumption are presented in **Table 2**. No group or sex differences were observed (*P* > 0.05).

**TABLE 1:** General characteristics of participants in long-chain n–3 PUFA and placebo groups participating in an 18-wk resistance exercise intervention^1^

	Placebo				Long-chain n–3 PUFA			
	Men (*n* = 13)		Women (*n* = 10)		Men (*n* = 14)		Women (*n* = 13)	
	Baseline	18 wk	Baseline	18 wk	Baseline	18 wk	Baseline	18 wk
Age, y	71.5 ± 5.1		70.9 ± 2.6		69.8 ± 4.0		70.5 ± 3.9	
Height, cm	171.6 ± 8.0		160.1 ± 6.7		177.1 ± 4.6		160.9 ± 7.0	
Body mass, kg	73.2 ± 11.5	72.5 ± 11.3	66.0 ± 12.7	65.2 ± 13.0	78.6 ± 16.7	78.7 ± 16.9	66.4 ± 12.4	67.3 ± 13.3
BMI, kg/m^2^	24.7 ± 2.6	24.4 ± 2.6	25.8 ± 4.6	25.4 ± 4.9	25.1 ± 5.3	25.1 ± 5.4	25.9 ± 4.9	26.3 ± 5.1
HR, bpm	66.8 ± 8.8	59.1 ± 10.3	73.7 ± 7.8	71.4 ± 12.7	65.9 ± 12.2	63.1 ± 16.7	68.8 ± 13.2	66.9 ± 0.2
BP, mm Hg								
Systolic	148.2 ± 20.8	137.1 ± 17.4	140.6 ± 12.9	127.1 ± 10.0	157.7 ± 19.4	138.1 ± 14.36	144.5 ± 21.4	139.6 ± 19.1
Diastolic	85.6 ± 10.2	77.5 ± 7.6	89.1 ± 9.0	81.5 ± 8.6	94.2 ± 9.7	83.3 ± 11.2	90.5 ± 16.7	85.1 ± 14.3

1 All values are means ± SDs, *P* < 0.05. There were no significant differences between placebo and treatment groups within sex at baseline, and no significant group, sex, or interaction (group by sex) effects were observed for any of the variables measured. BP, blood pressure; HR, heart rate.

**TABLE 2:** Baseline physical activity levels and weekly oily and nonoily fish consumption in long-chain n–3 PUFA and placebo groups participating in an 18-wk resistance exercise intervention^1^

	Placebo		Long-chain n–3 PUFA	
	Men(*n* = 13)	Women (*n* = 10)	Men (*n* = 14)	Women (*n* = 13)
Activity intensity, h/wk				
Light	6.8 ± 8.1	6.7 ± 5.5	6.9 ± 7.0	7.7 ± 9.4
Moderate	1.8 ± 1.8	0.8 ± 1.1	2.0 ± 1.4	1.2 ± 2.0
High	0.5 ± 1.3	0.4 ± 1.3	0.9 ± 1.9	0.8 ± 1.8
Fish consumption,^2^ portions/wk				
Oily	1.2 ± 0.8	1.1 ± 0.7	1.2 ± 0.8	1.1 ± 0.8
Nonoily	1.3 ± 0.9	1.4 ± 0.5	1.2 ± 0.6	1.4 ± 0.7

1 All values are mean ± SDs, *P* < 0.05. No significant group or sex or effects were observed for any of the variables measured.

2 One portion of fish = 140 g.

### Protocol adherence and effect of treatment on participant characteristics

Both the long-chain n–3 PUFA and placebo groups fully completed their exercise training sessions. Nineteen participants went on holiday for 5 ± 5.9 d, including 3.5 ± 1.7 training sessions; the number of holidays was not different between the 2 groups, and the duration of training was extended accordingly to make up the missed sessions. The ANOVA found no sex, group, or interaction effects (*P* > 0.05) for body mass, BMI (in kg/m^2^), resting HR, or systolic or diastolic BP.

### Knee-extensor isometric and isokinetic strength and SPPB

Data for maximal isometric torque, maximal isokinetic torque (at 30, 90, and 240°s^−1^), 4-m walk time, and chair-rise time are presented in **Table 3**. All participants were able to complete the 3 balance tests for the full 10 s, so these data are not presented. No group, sex, or interaction effects were seen for maximal isokinetic torque, 4-m walk time, or chair-rise time (*P* > 0.05). For maximal isometric torque, although no group or sex effects were observed (*P* > 0.05), a sex-by-group interaction was observed (*P* < 0.05). In men, the increase in maximal isometric torque after the 18-wk intervention was similar (*P* > 0.05) between groups: 41.8% ± 26.6% in the placebo group and 33.8% ± 30.7% in the long-chain n–3 PUFA group. However, in women, the increase after the 18-wk intervention in maximal isometric torque was greater (*P* < 0.05) in the long-chain n–3 PUFA group (34.3% ± 17.8%) than the placebo group (15.8% ± 10.6%).

**TABLE 3:** Knee-extensor maximal isometric and isokinetic torque and 4-m walk and chair-rise time in participants from long-chain n–3 PUFA and placebo groups participating in an 18-wk resistance exercise intervention^1^

	Placebo				Long-chain n–3 PUFA			
	Men (*n* = 13)		Women (*n* = 10)		Men (*n* = 14)		Women (*n* = 13)	
	Baseline	18 wk	Baseline	18 wk	Baseline	18 wk	Baseline	18 wk
Torque, N·m								
Maximal isometric	109.2 ± 33.9	152.5 ± 44.3	76.6 ± 15.3	88.1 ± 17.2	125.1 ± 35.8	164.3 ± 41.4	79.6 ± 10.6	103.5 ± 10.1*
Maximal isokinetic at 30°·s^𢄡^	131.9 ± 26.2	147.1 ± 38.4	83.5 ± 14.6	88.8 ± 17.3	145.1 ± 36.1	161.0 ± 37.5	92.0 ± 14.5	105.5 ± 8.6
Maximal isokinetic at 90°·s^𢄡^	84.1 ± 21.4	92.3 ± 22.2	52.6 ± 13.3	55.9 ± 16.5	102.5 ± 29.8	112.8 ± 30.1	56.6 ± 14.8	66.4 ± 14.7
Maximal isokinetic at 240°·s^𢄡^	45.7 ± 11.4	55.6 ± 14.6	28.2 ± 7.9	33.8 ± 9.4	61.0 ± 15.8	66.4 ± 14.4	34.1 ± 6.9	41.1 ± 7.6
4-m walk time, s	2.22 ± 0.25	1.99 ± 0.28	2.43 ± 0.41	2.25 ± 0.33	2.12 ± 0.27	1.87 ± 0.28	2.36 ± 0.28	2.20 ± 0.37
Chair-rise time, s	7.32 ± 1.10	6.35 ± 1.35	7.83 ± 1.96	6.71 ± 2.11	6.82 ± 1.40	5.80 ± 1.10	8.22 ± 1.51	6.60 ± 1.67

1 All values are means ± SDs, *P* < 0.05. The data at 18 wk were compared via an ANOVA for group, sex, and sex-by-group interaction effects with baseline values of the outcome measure as a covariate. *Significant interaction effect (group by sex), with the baseline-adjusted 18-wk value greater in the long-chain n–3 PUFA groups than in the placebo groups.

### Muscle ACSA, fat, and quality

Data for muscle ACSA, fat, and quality are presented in **Table 4**. No group, sex, or interaction effects were seen for muscle ACSA or fat (*P* > 0.05). An analysis of muscle quality data found no group or sex effects (*P* > 0.05), but an interaction effect (*P* < 0.05) was observed. The 18-wk intervention resulted in a similar increase in muscle quality in the placebo (36.6% ± 24.6%) and long-chain n–3 PUFA (31.0% ± 29.3%) groups in men. In women, the 18-wk intervention resulted in a greater increase in the long-chain n–3 PUFA group (27.0% ± 17.1%) than in the placebo group (8.8% ± 17.6%).

**TABLE 4:** MRI data from participants in long-chain n–3 PUFA and placebo groups participating in an 18-wk resistance exercise intervention^1^

	Placebo				Long-chain n–3 PUFA			
	Men (*n* = 12)		Women (*n* = 10)		Men (*n* = 13)		Women (*n* = 10)	
	Baseline	18 wk	Baseline	18 wk	Baseline	18 wk	Baseline	18 wk
Muscle ACSA, cm^2^	56.7 ± 8.7	58.8 ± 8.6	37.1 ± 5.7	37.8 ± 6.2	66.3 ± 4.1	67.9 ± 4.8	37.6 ± 6.1	39.4 ± 5.7
Muscle fat, %	8.24 ± 2.00	7.98 ± 1.95	8.84 ± 2.14	8.93 ± 2.14	7.28 ± 1.47	7.02 ± 1.46	8.80 ± 2.43	8.68 ± 2.14
Muscle quality, N·m/cm^2^	1.93 ± 0.49	2.58 ± 0.58	2.11 ± 0.55	2.22 ± 0.42	1.94 ± 0.53	2.47 ± 0.58	2.08 ± 0.36	2.62 ± 0.29*

1 All values are means ± SDs, *P* < 0.05. The data at 18 wk were compared via an ANOVA for group, sex, and sex-by-group interaction effects with baseline values of the outcome measure as a covariate. *Significant interaction effect (group by sex), with the baseline-adjusted 18-wk value greater in the long-chain n–3 PUFA groups than in the placebo groups. ACSA, anatomic cross-sectional area.

### Blood and muscle analysis

Data from the blood variables are presented in **Table 5**. No group, sex, or interaction effects were observed (*P* > 0.05) for glucose, insulin, TNF-α, or IL-6. A group but not sex or interaction effect (*P* < 0.05) was observed for plasma TG and erythrocyte EPA and DHA concentrations. For plasma TG concentrations, there were 3.9% ± 20.9% and 10.5% ± 30.7% increases in the men and women’s placebo groups, respectively. However, in the long-chain n–3 PUFA groups, there were 8.0% ± 20.8% and 17.7% ± 17.8% decreases in men and women, respectively. Erythrocyte EPA increased by 20.9% ± 71.2% and 89.6% ± 152.9% in the men and women’s placebo groups, respectively. In the long-chain n–3 PUFA groups, the increase in EPA was 232.1% ± 102.1% in men and 371.7% ± 227.2% in women. Erythrocyte DHA was 0.5% ± 15.4% and 8.7% ± 17.5% higher in the men and women’s placebo groups, respectively, after 18 wk. In the long-chain n–3 PUFA groups, the increase in DHA was 9.8% ± 14.5% in men and 33.8 ± 24.0% in women. Data from the muscle samples are presented in **Supplemental Figure 1** and **Supplemental Results**.

**TABLE 5:** Plasma measures of glucose, insulin, TGs, TNF-α, and IL-6 in participants in long-chain n–3 PUFA and placebo groups participating in an 18-wk resistance exercise intervention^1^

	Placebo				Long-chain n–3 PUFA			
	Men (*n* = 11)		Women (*n* = 9)		Men (*n* = 13)		Women (*n* = 12)	
	Baseline	18 wk	Baseline	18 wk	Baseline	18 wk	Baseline	18 wk
Glucose, mmol/L	5.33 ± 0.489	5.14 ± 0.34	6.07 ± 1.44	6.21 ± 1.64	5.21 ± 0.48	5.32 ± 0.64	5.80 ± 1.12	5.49 ± 1.20
TGs, mmol/L	0.86 ± 0.22	0.89 ± 0.32	1.29 ± 0.87	1.03 ± 0.32	1.00 ± 0.49	0.84 ± 0.34	0.88 ± 0.27	0.69 ± 0.16
Insulin, mU/mL	6.11 ± 2.66	5.71 ± 2.52	6.96 ± 4.67	6.02 ± 3.18	7.44 ± 4.91	7.78 ± 5.12	5.33 ± 3.04	5.5 ± 3.83
IL-6, pg/mL	1.34 ± 0.66	0.88 ± 0.24	1.77 ± 1.11	1.96 ± 1.02	1.32 ± 1.17	1.25 ± 0.75	1.63 ± 2.24	1.43 ± 1.96
TNF-α, pg/mL	4.91 ± 2.46	4.62 ± 2.05	4.81 ± 2.13	4.67 ± 1.89	4.95 ± 1.99	4.60 ± 1.73	5.27 ± 2.35	5.15 ± 2.21
EPA, % total lipids	1.44 ± 0.97	1.50 ± 1.33	1.47 ± 0.71	1.74 ± 0.50	1.47 ± 0.58	4.32 ± 1.64*	1.35 ± 0.79	4.68 ± 2.40*
DHA, % total lipids	5.16 ± 1.18	5.26 ± 1.40	5.18 ± 0.81	5.82 ± 0.62	5.89 ± 0.77	6.40 ± 0.68*	5.54 ± 1.95	6.52 ± 0.97*

1 All values are means ± SDs, *P* < 0.05. The data at 18 wk were compared via an ANOVA for group, sex, and sex-by-group interaction effects with baseline values of the outcome measure as a covariate. *Significant group effect with the baseline-adjusted 18-wk value higher in the long-chain n–3 PUFA groups than in their respective placebo groups. TG, triglyceride.

## DISCUSSION

Our results show, for the first time to our knowledge, that long-chain n–3 PUFA supplementation enhances the increases in maximal isometric torque and muscle quality after 18 wk resistance exercise training in older women but not in older men in a well-designed, double-blind, randomized placebo-controlled trial. These observations occur independently of changes in muscle mass. Rodacki et al. (18) demonstrated that long-chain n–3 PUFA supplementation (0.4 g EPA/d and 0.3 g DHA/d) enhanced the increases in maximal torque, the rate of torque development, and chair-rising performance after strength training for 90 d. This study was conducted only in women and was not placebo-controlled. Moreover, muscle mass was not quantified. Thus, our study is the first to our knowledge to show robustly that long-chain n–3 PUFA supplementation results in greater resistance exercise training–induced increases in muscle strength and quality in older women but no such effect in older men. Previous work (29) has suggested that the anti-inflammatory properties of long-chain n–3 PUFAs might ameliorate elevations in IL-6 and TNF-α, which have been shown to correlate with functional disability (30), and that these changes may underlie the beneficial effects of long-chain n–3 PUFAs in muscles. In our study, however, we did not detect any differences in plasma TNF-α or IL-6 between the groups postintervention, in agreement with reports from other studies (15, 16).

Taken together, the results of studies in this area raise some interesting questions regarding the mechanisms underlying these improvements. When long-chain n–3 PUFAs are consumed and the participants (mix of men and women) maintain habitual activity, mass and function increase to a similar extent (small increase in muscle quality) (17). However, in our study, when combined with resistance exercise there is a clear effect on function with little, if any, effect on mass (∼3-fold increase in muscle quality). This indicates that the mechanisms underlying the effects of long-chain n–3 PUFAs may be different dependent on exercise status. When habitual activity is maintained, the increases in muscle mass and function are likely because of, at least partly, increases in MPS (15, 16). We observed no effects of long-chain n–3 PUFAs on MPS. Although there were limitations in these data, such as not having an MPS measurement at the beginning of study and the small numbers of participants that precluded stratification by sex, the data are in agreement with the lack of effect of long-chain n–3 PUFAs on muscle mass. The main observation in this study was that long-chain n–3 PUFAs enhanced the increases in muscle quality after resistance exercise training in older women but not in men.

Long-chain n–3 PUFAs may improve muscle quality via reductions in intermuscular fat, which is known to influence muscle function independently of size (4). In this study, we found no effect of long-chain n–3 PUFA supplementation on intermuscular fat in either men or women, in agreement with previous research (17, 31). It is worth noting that this study did not measure intramuscular fat content or indeed the species of fat. Neuromuscular function, which decreases with age (32–35), also contributes to the loss of muscle strength and quality in older adults (36, 37). There is evidence that long-chain n–3 PUFA supplementation can improve neuromuscular function in young healthy volunteers (38), which may be mediated by DHA, an essential constituent of membrane phospholipids (39). Indeed, DHA is involved in numerous neural functions such as receptor affinity, modulation of signal transduction, and cell membrane fluidity (40); to our knowledge, however, its effects have not yet been explored in older adults.

Moreover, single muscle fiber contractile properties, especially force generation and contractile speed, decrease with age and are associated with the risk of falling, particularly among women (41). In fact, in old type II muscle fibers, a decrease in specific force (i.e., force per unit cross-sectional area) and Ca^2+^ sensitivity is seen in both type I and type II fibers (42). Whether long-chain n–3 PUFAs can improve contractile properties of skeletal muscle fibers independently of changes in mass remains to be established in older adults. In cardiomyocytes, it has been shown that long-chain n–3 PUFAs improve contractile function after β- and α-adrenergic stimulation (43). In addition, in studies of hamster left ventricular papillary muscles, long-chain n–3 PUFAs, when administered alongside propionyl-l-carnitine and the coenzyme Q10, increased cardiac function via increases in shortening velocity and force and power generation without an increase in fiber size (44). Furthermore, in rat diaphragms, EPA has been reported to preserve specific force via the attenuation of calpain activation after endotoxin injection (45). Although there are currently no data to our knowledge in older adults that demonstrate such effects, our data suggest that long-chain n–3 PUFAs, although participating in resistance exercise training may improve contractile function via improvements in neural function and intrinsic contractile properties of the muscle fibers independently of changes in size. However, why the effect of long-chain n–3 PUFAs on any of these suggested mechanisms would differ between sexes is not clear.

It has been proposed that these sex differences could be attributed to differences in the enrichment of long-chain n–3 PUFAs into cell membranes. Previous work has shown, at the same dose of long-chain n–3 PUFAs, a greater EPA and total long-chain n–3 PUFA enrichment in plasma phosphatidylcholine in women after fish-oil supplementation (8 wk with 0.7 g EPA/DHA or 1.8 g EPA/DHA) (46). However, in our study, the postsupplementation concentration of erythrocyte and muscle EPA and DHA was not different between men and women, although the variability was large. Another factor that potentially influenced our findings is a difference in nutritional intake between groups, which may have affected the adaptations to resistance exercise training. Apart from the fish diaries, which were not different between our groups, we did not collect any other data relating to habitual dietary intake. Sex differences in the response to resistance exercise itself may potentially explain the sex-specific effects of this study. As we have recently published (8), although others do not agree (7), older women do not increase muscle strength to the same magnitude as older men. It may be possible that long-chain n–3 PUFAs only have effects in women because there is a greater capacity for improvement; i.e., their normal response is suboptimal compared with men. This would make women more amenable to the effects of long-chain n–3 PUFAs. These mechanisms proposed are currently speculative, and further work is clearly needed to investigate them.

Plasma TGs over the 18-wk resistance exercise training intervention decreased to a greater extent in the long-chain n–3 PUFA group, as has been widely reported in the literature (47). No differences in fasting glucose and insulin were found between long-chain n–3 PUFA and placebo groups in this study, in agreement with a meta-analysis in this area (48).

Although our study found benefits of long-chain n–3 PUFAs on muscle function in older women, this was not reflected in changes in functional abilities. However, it is important to emphasize that the latter was not the outcome on which the study was powered. Although both long-chain n–3 PUFA and placebo groups improved SPPB scores after the 18-wk intervention, there were no significant differences between groups in either men or women. This might be because of the fact that muscle power is more important than strength for these measures of daily activities (49). Indeed, we did not find any changes in isokinetic torque. In conclusion, we have demonstrated for the first time to our knowledge that 3 g long-chain n–3 PUFAs/d enhances the resistance exercise training–induced increases in muscle quality and maximal isometric torque in older women but not older men. The findings of a benefit in women are of particular importance because women live longer than men and, because of their lower starting strength, cross the disability threshold earlier (50). Muscle ACSA, acute measures of MPS and p70s6k activity, intermuscular fat, and functional abilities were not altered by long-chain n–3 PUFA supplementation, although the study was not powered sufficiently to determine sex differences in the responses of all of these variables. Further work is warranted to investigate these differences.

## Footnotes

14 Abbreviations used: ACSA, anatomic cross-sectional area; BP, blood pressure; D_2_O, deuterated water; HR, heart rate; MPS, muscle protein synthesis; SPPB, short-performance physical battery; TG, triglyceride.
